# Translational coupling of neighboring genes in prokaryotes

**DOI:** 10.1128/jb.00255-25

**Published:** 2025-09-08

**Authors:** Kerry M. Brown, Joseph T. Wade

**Affiliations:** 1 Wadsworth Center, New York State Department of Health, Albany, New York, USA; 2 Department of Biomedical Sciences, College of Integrated Health Sciences, University at Albany, SUNY, Albany, New York, USA; 3 RNA Institute, University at Albany, SUNY, Albany, New York, USA; The Ohio State University, Columbus, Ohio, USA

**Keywords:** translational coupling, translation

## Abstract

Prokaryotic genomes are gene-dense, so genes in the same orientation are often separated by short intergenic sequences or even overlap. Many mechanisms of regulation depend on open reading frames (ORFs) being spatially close to one another. Here, we describe one such mechanism, translational coupling, where translation of one gene promotes translation of a co-oriented neighboring gene. Translational coupling has been observed across the prokaryotic kingdom. Coupling is most efficient when the intergenic distance between ORFs is small. Coupling efficiency is influenced by RNA secondary structure, the presence of a Shine-Dalgarno (SD) sequence, and potentially by other *cis*-acting elements. While the mechanism of translational coupling has not been firmly established, two models have been proposed. In the RNA unfolding model, translation of the upstream gene in a pair disrupts inhibitory RNA secondary structure around the start codon of the downstream gene. Alternatively, the reinitiation model proposes that the same ribosome—either the 30S or complete 70S—translates both genes in a coupled pair. We describe evidence in support of each model, and we discuss the functional implications of translational coupling.

## NEIGHBORING GENES IN PROKARYOTES ARE OFTEN COORDINATELY EXPRESSED

Prokaryotic genes are arranged in operons, with functionally related genes often located adjacent to one another (1). There are several ways in which the operonic organization of genes facilitates the coordination of their expression. First, genes within an operon are transcribed as a single mRNA, meaning that the regulation of transcription initiation will impact all genes in the operon equally (1). Second, if an upstream gene in an operon is translationally silenced, either by mutation or through regulation, premature transcription termination prevents expression of downstream genes in the operon, a phenomenon known as “polarity” (2, 3). In bacteria, polarity is due to the Rho transcription termination factor, which loads onto untranslated RNA (4). In archaea, polarity is likely due to the FttA transcription termination factor (5). Third, because of the effects of the ribosome on RNA secondary structure, translation of upstream genes can impact Rho-independent termination of downstream genes in an operon (4). Together, these mechanisms ensure that the expression of functionally related genes is often coordinated by virtue of their position as neighbors in the genome. Here, we discuss an additional mechanism that coordinates the expression of neighboring genes: translational coupling. In translational coupling, translation of one gene promotes translation of a nearby downstream gene that is positioned in the same orientation (i.e., tandem gene pairs). Here, we describe what is known about translational coupling, the factors that affect it, and possible mechanisms.

## PROKARYOTIC ORFs ARE OFTEN CLOSELY SPACED

Prokaryotic genomes have a high gene density, with ~0.8–1.2 genes per kilobase of genomic DNA (6). Therefore, it is unsurprising that genes are often close to one another. Indeed, as many as a third of genes in prokaryotic genomes overlap (7), with the majority of overlaps being co-directional (8). We refer to closely spaced or overlapping, co-directional pairs of protein-coding genes as “spatially coupled” ORFs. In cases where spatially coupled ORFs overlap, most overlaps are short, with >80% of gene pairs sharing <30 bp with their neighbor (7). A large portion of overlaps comprise only a few base pairs, with a high frequency of cases where start and stop codons overlap (7, 9). Presumably, there is selective pressure against longer overlaps that, by definition, impose constraints on the encoded protein sequence of both overlapping genes.

**Fig 1:**
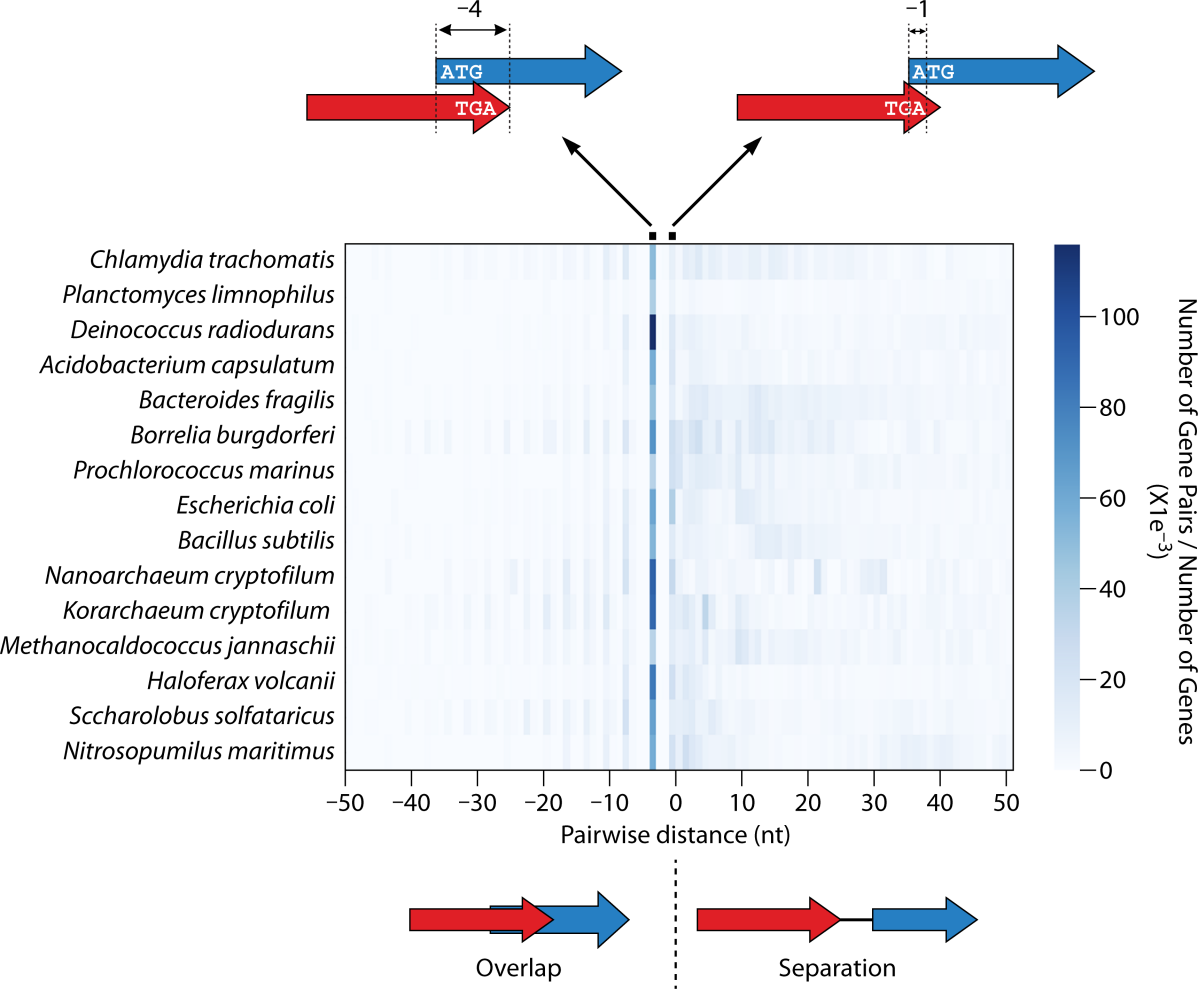
Distribution of distances between neighboring genes on the same strand. Heatmap showing the frequency of intergenic distances for co-oriented, neighboring genes in selected bacteria (*Chlamydia trachomatis*, *Planctomyces limnophilus*, *Deinococcus radiodurans*, *Acidobacterium capsulatum*, *Bacteroides fragilis*, *Borrelia burgdorferi*, *Prochlorococcus marinus*, *Escherichia coli*, and *Bacillus subtilis*) and archaea (*Nanoarchaeum equitans*, *Korarchaeum cryptofilum*, *Methanocaldococcus jannaschii*, *Haloferax volcanii*, *Saccharolobus solfataricus*, and *Nitrosopumilus maritimus*). The *x*-axis indicates the spacing between the stop codon of the upstream gene in a pair and the start codon of the downstream gene; negative values indicate overlap, and positive values indicate separation. The intensity of color indicates the relative number of gene pairs with that spacing. The most common spacings (−4 nt and −1 nt) are indicated above the heatmap.

The distribution of distances between spatially coupled ORFs has a strong bias toward certain spacing motifs, with the trends being similar across all bacterial and archaeal species (Fig. 1). By far the most common spacing motif consists of overlapping start/stop codons in an “NTGA” configuration, i.e., the start codon of the downstream gene (*NTG*) and the stop codon of the upstream gene (TGA) have two overlapping bases (*NTG*A) (7, 9). This motif is commonly referred to as having “−4 nt” spacing, reflecting the relative positions of the start and stop codons. To a lesser extent, start and stop codons with −1 nt spacing (i.e., TG*ATG* or TA*ATG*) or −8 nt spacing (e.g., *NTG*NNTGA) are enriched in overlapping gene pairs (9). With the exception of these three specific spacing motifs, spatially coupled ORFs are considerably more likely to be separated than overlapping (9). The enrichment of the −4 nt spacing architecture is striking, especially since it imposes a constraint on the identity of the second amino acid in the protein encoded by the downstream gene, because the first nucleotide of the second codon must be “A” (7). This means that the second codon can only encode one of seven amino acids. While −4 nt spacing is effective for the functional consequences of spatial coupling (see below), it is not exceptional in this regard. Indeed, several early mechanistic studies of spatially coupled genes focused on gene pairs with alternative spacings. The abundance of −4 nt spacing is likely due to the ease with which it can evolve, since a start codon provides three of the four required bases.

## SPATIALLY COUPLED ORFs CAN HAVE COUPLED TRANSLATION

When two ORFs are spatially coupled, translation of the upstream ORF often promotes translation of its downstream neighbor. This is broadly referred to as “translational coupling.” The impact of translational coupling on the expression of downstream genes can range over several orders of magnitude, depending on the gene pair. Translational coupling was first demonstrated for the *Escherichia coli trpEDCBA* operon by Oppenheim and Yanofsky (10). The *trpE* and *trpD* ORF stop/start codons exhibit −1 nt spacing. The authors showed that mutations abolishing translation of the upstream gene, *trpE*, negatively impacted *trpD* expression, independent of transcription. The effect on *trpD* expression was >10 times more than that on genes further downstream in the same operon. The authors concluded that translation of *trpE* promotes translation of *trpD*. Shortly afterward, Schümperli et al. demonstrated that *E. coli galK* is translationally coupled to its upstream neighbor, *galT* (11). The *galT*/*galK* stop/start codons exhibit +3 nt spacing. In reporter constructs, *galK* expression was substantially reduced when the *galT* stop codon was moved away from the *galK* start codon; the highest expression of *galK* was observed for the wild type, +3 nt spacing. A year later, Baughman and Nomura, studying the *E. coli* L11 ribosomal protein operon, demonstrated that deletions of the ribosome-binding site (RBS) of the upstream *rplK* ORF reduced translation of the downstream *rplA* (12). The *rplK*/*rplA* stop/start codons exhibit +3 nt spacing. Many subsequent studies have demonstrated translational coupling between pairs of spatially coupled ORFs. While most early studies of translational coupling focused on genes in *E. coli*, the phenomenon has been observed in a wide range of bacterial species (13–22), as well as the archaeon *Haloferax volcanii* (23), and chloroplasts (24).

## THE IMPORTANCE OF ORF-ORF SPACING FOR COUPLED TRANSLATION

Most early studies of translational coupling focused on individual gene pairs expressed in *E. coli*, some studies with naturally occurring pairs, and others with artificial constructs. The authors used reporter gene assays to investigate the impact of start/stop codon spacing on the efficiency of translational coupling. Three general trends emerged: (i) translational coupling is most efficient with spacings between −4 and +3 nt; (ii) as spacing is increased to ~+40 nt, translational coupling still occurs but with ~3- to 10-fold reduced efficiency; (iii) increases in overlap (i.e., more negative spacing) lead to a larger decrease in coupling efficiency than the equivalent increases in spacing (i.e., more positive spacing) (11, 14, 25–35).

A more recent study examined the impact of start/stop codon spacing on translational coupling for three naturally occurring unidirectional gene pairs from *E. coli* and three from the archaeal species *H. volcanii*, using similar reporter gene set-ups for each of the two organisms (9). The selected gene pairs have spacings between −4 and +1 nt. For both organisms, increases in spacing up to 30–40 nucleotides led to a gradual decrease in coupling efficiency, with spacings >30 nt showing little to no translational coupling, suggesting that translational coupling functions similarly in bacteria and archaea. However, the effect of spacing on coupling efficiency differed between the individual gene pairs within the same organism, suggesting that the specific sequence context influences translational coupling.

Three studies took a more systematic approach to studying translational coupling in *E. coli*, using artificial constructs that allowed for more rigorous analysis by maintaining the overall sequence context. Osterman et al. showed that varying spacing between −4 and +3 nt had no impact on coupling efficiency (36). Similarly, Tian and Salis showed that increasing spacing across a range from −4 to +25 nt had no impact on coupling efficiency; by contrast, increasing overlap, with spacing ranging from −4 to −25 nt, strongly decreased coupling efficiency in a distance-dependent manner, with no translational coupling observed for −25 nt spacing (37). However, the authors also observed much higher coupling efficiency for −4 nt spacing than any other spacing, inconsistent with other studies. Lastly, Levin-Karp et al. considered much larger spacings, ranging from +50 to +850 nt (38). The authors observed a gradual decrease in coupling efficiency from +50 to +350 nt, although translational coupling was observed at spacings considerably larger than those seen in other studies.

As described above, the trends in spacing requirements for translational coupling in *E. coli* are largely consistent across studies; however, there are some notable exceptions. Schoner et al. showed that moving from +3 to +5 nt spacing led to a ~50% drop in coupling efficiency, and moving to +13 nt spacing abolished translational coupling entirely (39). Similarly, Liljeström et al. showed that moving from −4 to +3 nt spacing led to a ~30 fold drop in coupling efficiency (40). This reinforces the idea that the specific sequence context influences the efficiency of coupling.

Most functional studies of translational coupling have used *E. coli* as a model system. However, two studies of translational coupling in Firmicutes*—Lactococcus lactis* and *Bacillus subtilis*—suggest that spacing requirements are more stringent than those in *E. coli*. Specifically, van de Guchte et al. showed in *L. lactis* that coupling is most efficient with −4 nt spacing, and coupling efficiency decreased progressively with each additional nucleotide increase in spacing from −4 to +3 nt; by contrast, the same reporter constructs assayed in *E. coli* showed little difference in coupling efficiency (14). Sprengel et al. demonstrated efficient coupling with −4 nt spacing in *B. subtilis* but ~10-fold lower coupling efficiency for +12 nt spacing. A similar trend was seen for a coupled gene pair from tobacco chloroplasts; using *in vitro* translation with a chloroplast extract, Yukawa et al. showed that a change from −10 nt spacing to +2 nt spacing caused an approximately twofold drop in coupling efficiency, and a change to +11 nt almost completely abolished translational coupling (24). Overall, studies of translational coupling from species other than *E. coli* suggest that while the phenomenon of coupling is widespread, the spacing parameters likely vary between species.

## OTHER FACTORS THAT INFLUENCE TRANSLATIONAL COUPLING

The extent of translational coupling does not necessarily correlate with the translation level of the upstream gene. A study of four coupled gene pairs in the *E. coli* chemotaxis pathway showed that varying the translation initiation rate of the upstream gene over a 30-fold range by altering the ribosome-binding site had no impact on translation of the downstream gene (41). Similarly, altering the translation elongation rate of the bacteriophage IKe gene V, or altering the number of ribosomes that complete translation of gene V, had little impact on translation of the downstream gene VII in *E. coli* (42). These data suggest that translational coupling can saturate as translation levels of the upstream gene increase. Consistent with this, Lindahl et al. (43) showed that, for the *E. coli rplC-rpsJ* gene pair, translation of *rplC* correlated with that of *rpsJ* for lower translation rates, but *rpsJ* translation saturated at higher rates of *rplC* translation. Lindahl et al. also showed that translational coupling is independent of the RNA polymerase, since translational coupling was observed for transcripts made by either *E. coli* RNA polymerase or T7 RNA polymerase (43).

Downstream genes in coupled pairs are associated with an SD sequence at least as often as the upstream gene (23). Several studies have experimentally examined the impact of an SD sequence for the downstream gene on translational coupling. An early study of translational coupling by Schoner et al. (39) showed that mutating the SD for the downstream gene in a coupled pair caused a large decrease in coupling efficiency. Similarly, the addition of an SD sequence to an artificial reporter construct for translational coupling led to an approximately threefold increase in expression that was largely due to translational coupling rather than independent translation of the downstream gene (35). The most extensive investigation of the importance of the SD for translational coupling was by Huber et al. (23), who studied four *E. coli* gene pairs and seven *H*. *volcanii* gene pairs. In no case was the SD absolutely required for translational coupling, but the SD did contribute to translational coupling for all four *E. coli* gene pairs and five of the seven *H*. *volcanii* gene pairs. The impact of mutating the SD varied considerably across individual gene pairs. Similarly, systematic studies using a bicistronic reporter in *E. coli* showed that removing an intergenic SD moderately decreased coupling efficiency (36), with the magnitude of the effect varying depending on the start/stop codon spacing. The same study showed that an appropriately positioned A/U-rich sequence—a known translation enhancer that likely binds ribosomal protein S1—can also moderately promote translation of the downstream gene in a translationally coupled pair (36).

To systematically identify *cis*-acting sequences that promote translational coupling, Andrè et al. introduced random G-less (to avoid creating an SD sequence) 12mer sequences immediately upstream of the stop codon in a coupled gene reporter construct with −1 nt spacing (44). After selecting for sequences that allow translational coupling, the authors sequenced 100 randomly selected clones. No strong sequence enrichments were identified, although there was a modest enrichment for A/T 6 bp upstream of the stop codon. Overall, these data suggest that there are no specific sequence features in the region around the stop/start codons that strongly influence translational coupling. Chemla et al. took a similar approach, screening a library of random sequences for those that inhibit translational coupling (45). The extent of predicted RNA secondary structure immediately downstream of the stop codon for the upstream gene correlated negatively with the efficiency of coupling. This is in contrast to repressive RNA secondary structure observed around the RBS for the downstream gene for many translationally coupled gene pairs (see below) but may indicate a position-dependent effect of RNA secondary structure on translational coupling.

## POSSIBLE MECHANISMS OF TRANSLATIONAL COUPLING: (I) RNA UNFOLDING

**Fig 2:**
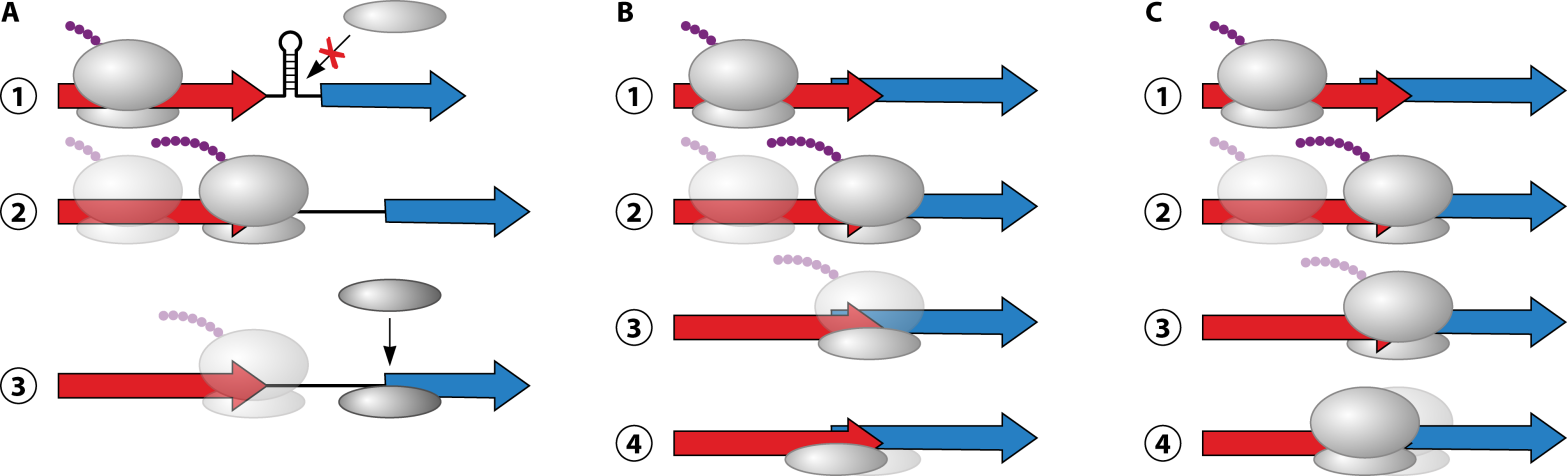
Models for translational coupling. (**A**) RNA unfolding model. (Step 1) RNA secondary structure prevents translation of the downstream gene. (Step 2) As the ribosome translates the upstream gene, it disrupts the RNA secondary structure, facilitating (Step 3) translation initiation of the downstream gene. (**B**) 30S scanning model. (Steps 1–3) After completing translation of the upstream gene, the ribosome releases the polypeptide and the 50S subunit dissociates. (Step 4) The 30S subunit remains associated with the RNA and scans until it reaches the start codon of the downstream gene, where it initiates translation. (**C**) 70S scanning model. (Steps 1–3) After completing translation of the upstream gene, the ribosome releases the polypeptide but remains associated with the RNA. (Step 4) The ribosome then scans until it reaches the start codon of the downstream gene, where it initiates translation.

Two mechanisms have been proposed for translational coupling: (i) an “RNA unfolding” model in which translation of the upstream gene disrupts RNA secondary structure around the RBS of the downstream gene, thereby activating canonical translation of the downstream gene (Fig. 2A); and (ii) a “ribosome scanning” model, in which the same ribosome (either the 30S alone or the 70S complex) that translates the upstream gene also translates the downstream gene (Fig. 2B and C). As discussed below, there is evidence for and against each model, and the two models are not mutually exclusive.

The RNA unfolding model (Fig. 2A) is closely analogous to transcription and translation attenuation mechanisms, where ribosome stalling at specific codons prevents the formation of regulatory hairpins by binding to RNA sequences that would otherwise form intramolecular base-pairs (46). Similarly, elongating/terminating ribosomes can prevent the formation of RNA hairpins that form transcription terminators (4). In translational coupling, effects on RNA structure could be due to occlusion of hairpin-forming sequences by ribosomes translating the upstream gene, or active unwinding of hairpins by ribosomes, which have inherent helicase activity (47). Several examples of coupled downstream genes were shown to be translationally silenced in the absence of coupling due to predicted or experimentally determined RNA secondary structure encompassing the RBS (48–54). In some cases, mutations predicted to disrupt the secondary structure cause increased expression of the downstream gene in the absence of translational coupling, consistent with the RNA unfolding model (17, 49–55). Indeed, genome-scale analysis of RNA structure suggests that translationally coupled gene pairs with inter-gene base-pairing are more likely to have matched translation levels (56). Thus, downstream genes in coupled pairs are often translationally silenced, and coupled translation can overcome this silencing. This ensures that the translation of the downstream gene is coordinated with that of its upstream partner.

RNA structure-based repression of downstream genes in translationally coupled pairs could be an indication of the RNA unfolding mechanism. However, it could also be due to evolutionary pressure to facilitate the dependence of translation on that of the upstream gene. Indeed, some downstream genes in coupled pairs are translationally silent in the absence of coupling due to an inherently weak affinity for 30S ribosomes rather than RNA secondary structure (30). Moreover, for translationally coupled genes that are closely spaced, it would not be possible for a ribosome to initiate translation of the downstream gene before the ribosome terminating translation of the upstream gene had dissociated from the RNA. Nonetheless, the RNA unfolding model is supported by additional evidence in the case of the bacteriophage f1 genes VII and IX that are coupled with a −4 nt spacing. Blumer et al. (48) predicted a long hairpin that ends a short distance upstream of gene IX and would likely prevent translation initiation in the absence of coupling. Changing the spacing to +62 nt by moving the gene VII stop codon upstream maintains translational coupling, which can be explained by the fact that the hairpin is still partly encompassed by the truncated gene VII. However, changing the spacing to +89 nt, which positions the gene VII stop codon well upstream of the hairpin, prevents translational coupling. The RNA unfolding model is also more likely in cases where the spacing or overlap is sufficiently large that a ribosome can initiate translation of the downstream gene while the ribosome translating the upstream gene is unfolding the RNA. For example, the bacteriophage MS2 coat and lysis genes exhibit -50 nt spacing, meaning that a ribosome terminating translation of the coat gene can prevent the formation of RNA secondary structure around the RBS of the lysis gene without physically occluding ribosome binding (51, 52). Similarly, the *E. coli rpmI-rplT* translationally coupled gene pair exhibits +52 nt spacing, meaning that a ribosome translating *rpmI* can prevent the formation of inhibitory secondary structure without occluding ribosome binding to the *rplT* RBS (27, 28).

## POSSIBLE MECHANISMS OF TRANSLATIONAL COUPLING: (II) RIBOSOME SCANNING

Several lines of evidence suggest that the RNA unfolding model cannot fully explain translational coupling in many cases. First, the ribosome initiating translation of the downstream gene would have to compete kinetically with refolding of the RNA following dissociation of the upstream ribosome. Second, as described above, the efficiency of coupling decreases as the distance between the stop/start codons of the coupled gene pair increases; distance increases of <20 nt can substantially reduce coupling efficiency but would not be expected to prevent the disruption of inhibitory RNA secondary structure around the downstream gene’s RBS in most cases. Third, translational coupling has been observed for gene pairs where there is no expected secondary structure around the RBS of the downstream gene (30, 33, 36, 55). Fourth, translation of downstream genes in coupled pairs in *E. coli* is more resistant to kasugamycin than that of other genes (57, 58). Since kasugamycin functions in part by inhibiting steps prior to formation of the 70S initiating ribosome (59), the reduced effect of kasugamycin on downstream genes in coupled pairs is most consistent with a ribosome scanning model involving the 70S ribosome (see below).

Early studies of translational coupling proposed an alternative mechanism: the ribosome scanning model, in which either the 30S subunit (Fig. 2B) or the complete 70S ribosome (Fig. 2C) remains on the RNA after completing translation of the upstream gene, scans along the RNA, and initiates translation on the start codon of the downstream gene (10, 11, 25, 40, 60). In this model, any secondary structure in the RNA around the RBS of the downstream gene would either be prevented from forming by the ribosome occluding hairpin-forming sequences or actively unfolded by the ribosome’s helicase activity. The first evidence for the ribosome scanning model came from a study of the bacteriophage *fr* coat and lysis genes, which are coupled with −38 nt spacing (61). If the lysis gene start codon is moved such that spacing changes to −50 nt, translational coupling is still observed. However, if start codons with both −38 nt and −50 nt spacing are included in the same construct, ribosomes exclusively initiate at the start codon with −38 nt spacing, i.e., the start codon closest to the stop codon of the upstream gene. Similarly, if the lysis gene start codon is moved such that spacing is either +35 nt or +23 nt, translational coupling is maintained in each case. But when both start codons are included in the same construct, ribosomes exclusively initiate at the start codon with +23 nt spacing, i.e., the start codon closest to the stop codon of the upstream gene. These data support a model in which a ribosome scans from the stop codon of the coat gene until it reaches a start codon, meaning that the start codon with the shorter stop-start distance will be selected for translation of the lysis gene.

The same study looked at the related coat-lysis gene pair from bacteriophage MS2, which has −50 nt spacing and had been proposed to use the RNA unfolding mechanism (see previous section) (51, 52). Including a second lysis gene start codon with −38 nt spacing leads to translation initiation from both start codons, consistent with the RNA unfolding model. However, when the coat gene stop codon was moved upstream, creating positive spacing, initiation favored the nearest lysis gene start codon. This suggests that when the coupled stop/start codons are closer together than in the wild-type sequence, translational coupling uses the ribosome scanning mechanism.

Additional evidence for the ribosome scanning model came from two studies that used so-called “specialized” ribosomes, where an additional ribosome was produced in *E. coli*, with the 16S rRNA modified to recognize a non-canonical SD sequence (54, 62). If the SD of the upstream gene in a coupled pair is altered to match the specialized ribosomes, translation of the downstream gene would also require a non-canonical SD for translation initiation to occur via ribosome scanning. Rex et al. used this approach to investigate the mechanism of translational coupling for *E. coli aphH-aptA* (54). While the data are consistent with the ribosome scanning mechanism, interpretation was confounded by a likely effect of mutating the SD sequence on the RNA secondary structure around the RBS. Govantes et al. used specialized ribosomes to investigate the mechanism of translational coupling for *Klebsiella pneumoniae nifL-nifA* expressed in *E. coli* (62). They were able to selectively inactivate canonical ribosomes using spectinomycin, while the specialized ribosomes carried a spectinomycin-resistance mutation. The authors modified the SD of the downstream gene, *nifA*, to recognize only specialized ribosomes. They then selectively produced specialized ribosomes by growing cells in the presence of spectinomycin. Under these conditions, efficient translation of *nifA* was only observed if the SD of the upstream gene, *nifL*, was also modified to recognize specialized ribosomes. These data provide strong evidence that the same ribosome that translates *nifL* also translates *nifA*, in support of the ribosome scanning model.

## 30S VS 70S SCANNING

Early work on translation by *E. coli* ribosomes demonstrated that the 30S subunit is the last to dissociate from the mRNA, leading to the suggestion that the 30S subunit could scan along the mRNA following translation termination (63). This led to the proposal of a 30S ribosome scanning mechanism for translational coupling (Fig. 2B) (10, 26). This is an attractive model because of striking parallels with translational coupling in the eukaryotic virus family *Caliciviridae*, where translational coupling has been proposed to occur via scanning of the small ribosomal subunit. Translational coupling in the *Caliciviridae* family frequently uses −1 or −4 nt spacing, and the efficiency of coupling decreases as intergenic spacing increases. Moreover, RNA secondary structure in the *Caliciviridae* upstream gene likely prevents coupling-independent translation initiation of the downstream gene (64). Most evidence suggests that only the small ribosomal subunit scans during translational coupling of *Caliciviridae* gene pairs (64), but a more recent study suggested that the full 80S ribosome can scan (65). One important difference between viral and prokaryotic translational coupling is the requirement for a specific *cis*-acting element toward the end of the upstream gene of the coupled pair in the *Caliciviridae*.

Evidence in support of the 30S scanning model for prokaryotes came from a study of the effects of the translation initiation factors IF2 and IF3 on translational coupling (35). This study showed that when translation of a downstream coupled gene initiates using a modified transfer RNA (tRNA) that is defective in binding IF2, the overexpression of IF2 increases expression of the downstream gene, suggesting that IF2 promotes translational coupling. Given that IF2 interacts initially with 30S ribosomal subunits, these data support a 30S scanning mechanism for translational coupling. In the same study, IF3 overexpression was shown to reduce coupling efficiency. The authors proposed that the inhibitory effect of IF3 on translational coupling was due to IF3 preventing the association of scanning 30S ribosomes with weakly interacting mRNA. While these data are consistent with a 30S scanning model for translational coupling, interpretation of the data is challenging since overexpressing translation initiation factors may lead to pleiotropic effects that influence coupling efficiency.

Ribosome recycling factor (RRF) functions together with EF-G to promote the splitting of the 70S ribosome into the 50S and 30S subunits (66). If translational coupling requires 30S subunit scanning, depletion of RRF would be expected to inhibit coupling by preventing the splitting of 70S ribosomes after they complete translation of the upstream gene in a coupled pair. RRF is essential, so studies of its function have relied upon depletion rather than gene deletion. Four studies measured translational coupling in *E. coli* following depletion of RRF. In all four studies, RRF depletion had no effect on coupling efficiency (33, 34, 67, 68). In one study, 40% of RRF protein was estimated to remain after depletion (34), leaving open the possibility that sufficient RRF remained to promote ribosome splitting. However, two studies included a functional assessment of RRF depletion (33, 67), and one study showed no effect of adding RRF on translational coupling using an *in vitro* translation system (68). Saito et al. showed that RRF depletion led to ribosome accumulation in 3′ UTRs and stacked upstream of stop codons, indicating that very little RRF activity remained (67). No effect of RRF depletion was observed on reporter gene constructs or any chromosomal coupled gene pair. Together, these studies argue against translational coupling requiring 30S ribosome scanning. Further evidence against 30S scanning came from Metelev et al., who reported that 50S and 30S subunits spend roughly the same amount of time on RNA in *E. coli* (69).

Saito et al. also argued that RRF depletion could impact translational coupling that involves a 70S ribosome scanning mechanism, reasoning that the increased levels of 70S ribosomes at stop codons could lead to increased coupling efficiency. The lack of an effect of RRF depletion on translational coupling therefore provides evidence against a 70S ribosome scanning mechanism, although, as discussed above, the efficiency of coupling can saturate at relatively low levels of translation for the upstream gene in a coupled pair (41–43).

As described earlier, the lower impact of kasugamycin on translation of downstream genes in coupled pairs is most consistent with a 70S ribosome scanning mechanism. More direct evidence in support of this mechanism comes from a study by Yamamoto et al., who investigated the ribosome scanning mechanism using an *in vitro* translation system with *E. coli* components (70). First, the authors provided evidence of ribosome scanning by showing that hybridization of a DNA oligonucleotide to the mRNA between a pair of coupled genes specifically reduced translation of the downstream gene. However, the spacing used was +73 nt, which most studies suggest would support only weak translational coupling *in vivo*. Next, the authors showed that translational coupling occurred when translation of the upstream gene was initiated with a 70S ribosome, with a template where translation of the downstream gene was not possible using a mixture of 30S and 50S subunits. These data support a 70S ribosome scanning mechanism rather than 30S. However, the same efficiency of coupling was observed with +1 nt and +39 nt spacing, inconsistent with most *in vivo* studies of translational coupling. Translational coupling in this assay was dependent upon IF3 and stimulated by IF1 in the presence of IF3. Consistent with a role for IF1 in 70S ribosome scanning, depletion of IF1 to 25% of wild-type levels caused a large reduction in coupling efficiency *in vivo*. Lastly, the authors showed that initiator tRNA could displace a tRNA from the P-site of a stalled ribosome, leading to scanning of the 70S ribosome to a nearby start codon, thus providing a possible mechanism for 70S ribosome scanning following termination of translation of the upstream gene in a coupled pair. However, scanning in this context was highly dependent upon the presence of an SD sequence, inconsistent with *in vivo* studies that demonstrated translational coupling in the absence of an SD sequence.

While this study provides strong evidence for a 70S ribosome scanning mechanism for translational coupling, it challenges the dogma for prokaryotic translation initiation, where decades of work have led to a detailed mechanistic understanding (71). Canonical initiation proceeds via assembly of a complex of the 30S ribosomal subunit with IF1, IF2, IF3, an initiator fMet-tRNA^fMet^, and a suitable mRNA, followed by recruitment of the 50S ribosomal subunit. IF1 functions in part to block the A-site of the 30S subunit, directing fMet-tRNA^fMet^ to the P-site. IF2 binds specifically to fMet-tRNA^fMet^, delivering it to the P-site. IF3 prevents the joining of the 50S ribosomal subunit until an appropriate fMet-tRNA^fMet^-mRNA interaction has been established. Yamamoto et al. reported that IF1 and IF3 enhance reinitiation by 70S scanning. This is counterintuitive because IF1 and IF3 canonically associate with the 30S subunit, with IF3 dissociating from the ribosome upon joining of the 50S subunit. Yamamoto et al. provided some evidence that addresses these apparent contradictions, reporting that both IF1 and IF3 associate directly with 70S ribosomes. A subsequent study described an interaction of IF3 with the 50S subunit in the context of the 70S ribosome, suggesting a function for IF3 bound to 70S ribosomes (72). Nonetheless, there are still many unanswered questions regarding a possible 70S scanning model for translational coupling, including the role of the initiation factors, the mechanism by which fMet-tRNA^fMet^ gains access to the P-site, and the mechanism that controls the specificity of translation initiation at an appropriate start codon.

There is evidence in support of both the RNA unfolding and the ribosome scanning models. While there is arguably more evidence in support of the ribosome scanning model, it requires a fundamental change in our understanding of translation initiation. The two mechanisms are not mutually exclusive, leaving open the possibility that either may be used, depending on the specific gene pair. Indeed, it is possible that both mechanisms could apply for the same gene pair, with some translation events for the downstream gene occurring by initiation with a new ribosome and some occurring by ribosome scanning.

## FUNCTIONAL CONSEQUENCES OF TRANSLATIONAL COUPLING

What are the functional implications of translational coupling? First, translational coupling facilitates the coordinated translational regulation of neighboring genes without the need for *cis*-acting elements. For example, the *E. coli rplK* gene is translationally repressed by ribosomal protein L1 (73), and regulation is “transferred” to the downstream gene *rplA* (encodes ribosomal protein L1) by translational coupling (12). Thus, translational coupling provides an additional mechanism to coordinate the expression of operonic genes. To ensure that translational regulation is coordinated between the two genes in a coupled pair, the downstream gene should not be expressed in the absence of translational coupling. This likely explains why many downstream genes in coupled pairs are translationally repressed when coupling is inactivated. Second, translational coupling may affect the expression of coupled genes in response to environmental stimuli. For example, kasugamycin differentially impacts the downstream genes in coupled pairs. Similar effects may be seen for other environmental factors that affect ribosome subunit assembly. Third, translational coupling has been suggested to promote co-translational protein folding, although no mechanism for this has been determined (74). Fourth, translational coupling has been proposed as an alternative way to initiate translation. Specifically, translation of a short upstream ORF—sometimes referred to as a uORF—could “deliver” a ribosome to the coupled downstream gene, meaning that the function of the short ORF is solely to drive translation of the downstream gene rather than encoding a functional protein (75). Ribosome profiling methods have uncovered large numbers of short ORFs (76), some of which have the potential for translational coupling with a downstream, annotated gene (75), so this may be a widespread mechanism. An interesting case where this phenomenon may have been exploited in nature is the mycobacterial interspersed repetitive units (MIRUs), short, repetitive sequences found in many mycobacteria. MIRUs appear to be mobile and insert themselves at start codons, such that an ORF within the MIRU becomes coupled to the gene at the insertion site with a −4 nt spacing. This likely facilitates insertion of the MIRU with minimal impact on expression of the gene where insertion occurs (77). There are likely to be additional functional consequences of translational coupling that are yet to be discovered. These may be useful for biotechnology applications. Indeed, the use of a short ORF to “deliver” a ribosome to a downstream gene has been exploited for biotechnology applications because the coupling architecture eliminates concerns about repressive RNA secondary structure (78).

## PERSPECTIVES

Translational coupling is an extremely common phenomenon in prokaryotes. For example, ~24%–30% of unidirectional gene pairs in *E. coli* and *H. volcanii* have been proposed to exhibit coupling based on their relative position (9). Although the phenomenon of translational coupling was first described nearly 50 years ago (10), its functional consequences and its mechanism(s) of action are still not fully understood. The large majority of mechanistic studies of translational coupling have focused on *E. coli*, potentially biasing our understanding of the process, especially given the extensive differences in translational mechanisms across the bacterial and archaeal kingdoms. The limited data on translational coupling in other species suggest that there are mechanistic differences, such as a more stringent spacing requirement for efficient coupling. Hence, future studies of translational coupling should consider a range of bacterial and archaeal species. Further work is needed to determine whether translational coupling occurs via an RNA folding mechanism, a ribosome scanning mechanism, or both.
